# Effects of exercise intervention parameters on anxiety symptoms in adolescents: a systematic review and meta-analysis of randomized controlled trials

**DOI:** 10.3389/fpsyg.2026.1893629

**Published:** 2026-09-23

**Authors:** Cheng Shiyue, Cao Dawei, Qi Yue, Shi Hongbo

**Affiliations:** 1 School of Physical Education, Huaibei Normal University, Huaibei, Anhui, China; 2 School of Football, Huaibei Normal University, Huaibei, Anhui, China; 3 Graduate School, Harbin Sport University, Harbin, Heilongjiang, China

**Keywords:** adolescents, anxiety disorder, exercise intervention, exercise parameters, meta-analysis

## Abstract

**Objective:**

This meta-analysis aimed to systematically evaluate the effects of exercise interventions (including aerobic exercise, Yoga, and Tai Chi) on anxiety symptoms in adolescents and to explore the moderating roles of intervention parameters.

**Methods:**

Randomized controlled trials (RCTs) examining the effects of exercise interventions on adolescent anxiety symptoms were searched in PubMed, Web of Science, CNKI, Wanfang Database, and VIP Information up to October 15, 2025. The Cochrane Risk of Bias tool was used for quality assessment. Meta-analysis, subgroup analyses, sensitivity analysis, and publication bias assessment were performed using Review Manager 5.4.

**Results:**

Twelve RCTs with a total of 1,874 participants were included. The overall effect size was small but statistically significant [SMD = −0.30, 95% CI (−0.60, −0.01), *P* = 0.04], with substantial heterogeneity (*I*^2^ = 87%, *P* < 0.00001). Subgroup analyses suggested that intervention effects may vary by exercise type, session duration, weekly frequency, and total intervention duration. Exploratory findings indicated that interventions with 30-min sessions, five times per week, and longer total duration (~45 weeks) were associated with larger reductions in anxiety symptoms. However, these subgroup findings should be interpreted cautiously due to the limited number of studies per subgroup.

**Conclusions:**

Exercise-based interventions may reduce anxiety symptoms in adolescents, although the effect magnitude is small and the evidence is limited by substantial heterogeneity. Exploratory subgroup analyses suggest that exercise parameters may influence intervention outcomes, but no definitive optimal prescription can be established based on the current evidence. Future research with standardized protocols and larger samples is warranted.

PROSPERO Registration: CRD420251265936

## 1 Introduction

Promoting the physical and mental health of teenagers and facilitating their all-round development is an important issue that the Central Committee of the Communist Party of China is concerned about, the people are concerned about, and all sectors of society are concerned about. With the rapid development of the economy and society, the environment in which teenagers grow up is constantly changing, and mental health problems are becoming increasingly prominent (Central People's Government of the People's Republic of China, 2023). Teenagers are the future of the country and the hope of the nation. As they grow up, they face complex and diverse challenges and are thus more prone to mental health problems (China Science and Technology Network, 2025). Among all kinds of mental health problems, anxiety is one that deserves particular attention (La Ossa et al., 2009). This negative emotional state—characterized by tension, unease, and worry—can put an individual in a state of physical tension and heightened alertness, often accompanied by mild disturbances in sleep and appetite, difficulty concentrating, irritability and depression. At all stages of life, individuals can be troubled by anxiety, and adolescence is a high-risk period (Yanhui and Rongxia, 2025) for anxiety disorders. Anxiety can interfere with teenagers' learning and daily life, prevent them from establishing smooth interpersonal communication, and have multiple negative impacts (Yilun et al., 2016) on their quality of life. Adolescence (10–19 years old) is a crucial period for an individual's physical and mental development, and mental health issues during this period are receiving widespread attention worldwide.

Research shows that regular scientific exercise can also bring many positive benefits (Guodong et al., 2025) to physical health. Exercise is widely regarded as a highly promising non-pharmaceutical therapy because of its low cost, high returns and low side effects. In recent years, an increasing number of studies have explored the improvement effect of exercise on adolescent anxiety, laying a solid foundation for continued research, but also suggesting that different types of exercise may have different neurophysiological mechanisms on anxiety, and their intervention effects should not be generalized. In other words, lumping up multiple forms of exercise for evaluation may mask the unique effects of specific types of exercise, such as “aerobic exercise,” and may not provide sufficient fine-grained evidence for the formulation of precise exercise prescriptions. Therefore, based on the consensus in the academic community, this study focuses on the perspective of aerobic exercise. First, at the theoretical mechanism level, aerobic exercise has been proven to effectively regulate the function of the hypothalamic-pituitary-adrenal axis, reduce cortisol levels at rest, and increase the release of brain-derived neurotrophic factor. These neurobiological changes are closely related to the relief of anxiety symptoms. Secondly, in terms of practical application, aerobic exercise has the characteristics of easy quantification and monitoring of intensity, high safety, and relatively low requirements for venues and equipment, making it easier to promote on a large scale and persist in the long term among adolescents. However, despite the wide application of aerobic exercise, there is a high degree of heterogeneity in exercise parameters for adolescent anxiety, which leads to an unclear “optimal dose” and makes it difficult to form unified clinical guidance recommendations.

Based on this, the present study employed a meta-analytic approach to systematically synthesize the findings from randomized controlled trials examining the effects of exercise interventions—including aerobic exercise, Yoga, and Tai Chi—on anxiety symptoms in adolescents. In contrast to previous meta-analyses that either broadly defined “exercise” without clear differentiation or simply compared multiple exercise types without further analysis of specific parameters, this study focused on addressing two key issues. First, we precisely defined the scope of the review: aerobic exercise, Yoga, and Tai Chi were all included within the analytical framework, but were strictly distinguished as three independent subgroups during subgroup analyses. This approach minimized the risk of research bias arising from confounding factors. Second, we refined the analysis of exercise parameters: in addition to evaluating the overall effect of exercise interventions on adolescent anxiety, we specifically examined the moderating effects of different parameters—including exercise type, session duration, weekly frequency, and total intervention cycle—on intervention outcomes. Our aim was to provide more specific and practical theoretical references for school-based physical health programs and psychological interventions, thereby offering evidence-based guidance for adolescents to improve their mental health through scientifically informed exercise participation.

## 2 Data sources and methods

### 2.1 Literature retrieval

Searches were conducted through databases such as China National Knowledge Infrastructure (CNKI), PubMed, and Web of science. The search period was from the time of database establishment to the search date (October 15, 2025). The Chinese database search terms are subject terms such as “aerobic exercise,” “running,” “swimming,” “skipping rope,” “dancing,” “teenagers” and “anxiety,” and the English database search formulas are shown in Table 1. A preliminary search yields 2,494 documents, including 226 in Chinese and 2, 206 in English.

**Table 1:** Search formula for literature.

Database	Retrieval formula
China National Knowledge Infrastructure	(Aerobic exercise OR aerobic training OR Tai Chi OR yoga) AND (teenage OR middle school OR student OR youth) AND (anxiety OR anxiety symptoms OR anxiety mood) AND (control group OR randomized controlled trial OR experimental group OR experimental group)
PubMed	(“Aerobic exercise”[MeSH Terms] OR “Aerobic exercise”[All Fields] OR “Aerobic Training”[All Fields] OR “Tai Chi”[MeSH Terms] OR “Tai Chi”[All Fields] OR “Tai Chi Chuan”[All Fields] OR Yoga[MeSH Terms] OR Yoga[All Fields]) AND (Adolescents[MeSH Terms] OR Adolescents[All Fields] OR Teenagers[All Fields] OR “School Students”[All Fields] OR Youth[MeSH Terms] OR Youth[All Fields]) AND (Anxiety[MeSH Terms] OR Anxiety[All Fields] OR “Anxiety Symptoms”[All Fields] OR “Anxious Mood”[All Fields]) AND (“control group”[All Fields] OR RCT[All Fields] OR “experimental group”[All Fields] OR “randomized controlled trial”[pt] OR “randomized controlled trial”[All Fields])
Web of science	(“Aerobic exercise”[MeSH Terms] OR “Aerobic exercise”[All Fields] OR “Aerobic Training”[All Fields] OR “Tai Chi”[MeSH Terms] OR “Tai Chi”[All Fields] OR “Tai Chi Chuan”[All Fields] OR Yoga[MeSH Terms] OR Yoga[All Fields]) AND (Adolescents[MeSH Terms] OR Adolescents[All Fields] OR Teenagers[All Fields] OR “School Students”[All Fields] OR Youth[MeSH Terms] OR Youth[All Fields]) AND (Anxiety[MeSH Terms] OR Anxiety[All Fields] OR “Anxiety Symptoms”[All Fields] OR “Anxious Mood”[All Fields]) AND (“control group”[All Fields] OR RCT[All Fields] OR “experimental group”[All Fields] OR “randomized controlled trial”[pt] OR “randomized controlled trial”[All Fields])

### 2.2 Criteria for inclusion and exclusion of literature

#### 2.2.1 Study design and language restriction

Only randomized controlled trials (RCTs) published in English or Chinese were included.

#### 2.2.2 Research subjects

Adolescents with anxiety (aged 10 to 19 years) who meet the diagnostic criteria for anxiety in the Diagnostic and Statistical Manual of Mental Disorders (DSM-4 or DSM-5) or the International Statistical Classification of Diseases and Related Health and Problems (ICD-10), or based on a valid scale, More commonly used effective scales include The Generalized Anxiety Disorder-7 (GAD-7) and the State-Trait Anxiety Inventory Scales such as STAI and the Depression Anxiety Stress Scale-21 (DASS-21) assess adolescents who have developed anxiety symptoms.

#### 2.2.3 Classification of interventions

The interventions included in this study were classified into the following three categories:

(1) Aerobic Exercise: rhythmic physical activity that continuously supplies energy through aerobic metabolism and involves large muscle groups of the body (Chen et al., 2017). Typical forms include running, swimming, rope skipping, and aerobic dance.

(2) Yoga: a typical mind-body exercise that combines slow voluntary body movements, full-body stretching and relaxation, abdominal breathing exercises, and focused meditation. Although it includes aerobic components, its core mechanisms involve mind-body regulation; therefore, this study treated Yoga as an independent subgroup.

(3) Tai Chi: a traditional Chinese mind-body exercise that integrates martial arts, health promotion, and cultural elements. It includes aerobic metabolic components and abdominal breathing training. This study also treated Tai Chi as an independent subgroup.

Classification basis: during the data extraction and analysis stage, the research team strictly categorized studies into the “aerobic exercise group,” “Yoga group,” or “Tai Chi group” based on the descriptions of intervention measures in the original literature. Through subgroup analyses, we compared the improvement effects of these three intervention types on anxiety symptoms and separately examined the moderating effects of exercise parameters within each type.

#### 2.2.4 Outcome measures

The assessment tools were scales measuring the severity of anxiety in adolescents. The main ones are the State-Trait Anxiety Inventory. STAI, Symptom Checklist 90, HADS Hospital Anxiety and Depression Scale, Zung Self-Rating Depression Scale, BDI-13 Baker Depression Scale (13 items), etc. Primary outcome measure: score on a scale assessing the severity of anxiety in adolescents. While tools like the HLBS and PHCSCS are not exclusively anxiety-specific scales, we only extracted and pooled data from their subscales or items that directly measured anxiety-related symptoms (e.g., the anxiety subscale of the SCL-90). This approach was adopted to ensure all pooled data directly reflected the outcome of interest.

#### 2.2.5 Exclusion criteria

References for which the original or raw data cannot be obtained; (1) Re-published literature with unclear expression of experimental data or analytical methods; (2) Conference papers, dissertations; (3) Non-randomized controlled trials; (4) References that do not conform to the subject of the article; (5) Literature with unavailable full text or inaccessible original data.

### 2.3 Literature screening

After removing duplicates from the documents using Endnote 20.0 software (Clarivate Analytics, Philadelphia, PA, USA), the documents were screened according to the inclusion and exclusion criteria listed, as shown in Figure 1.

**Figure 1:**
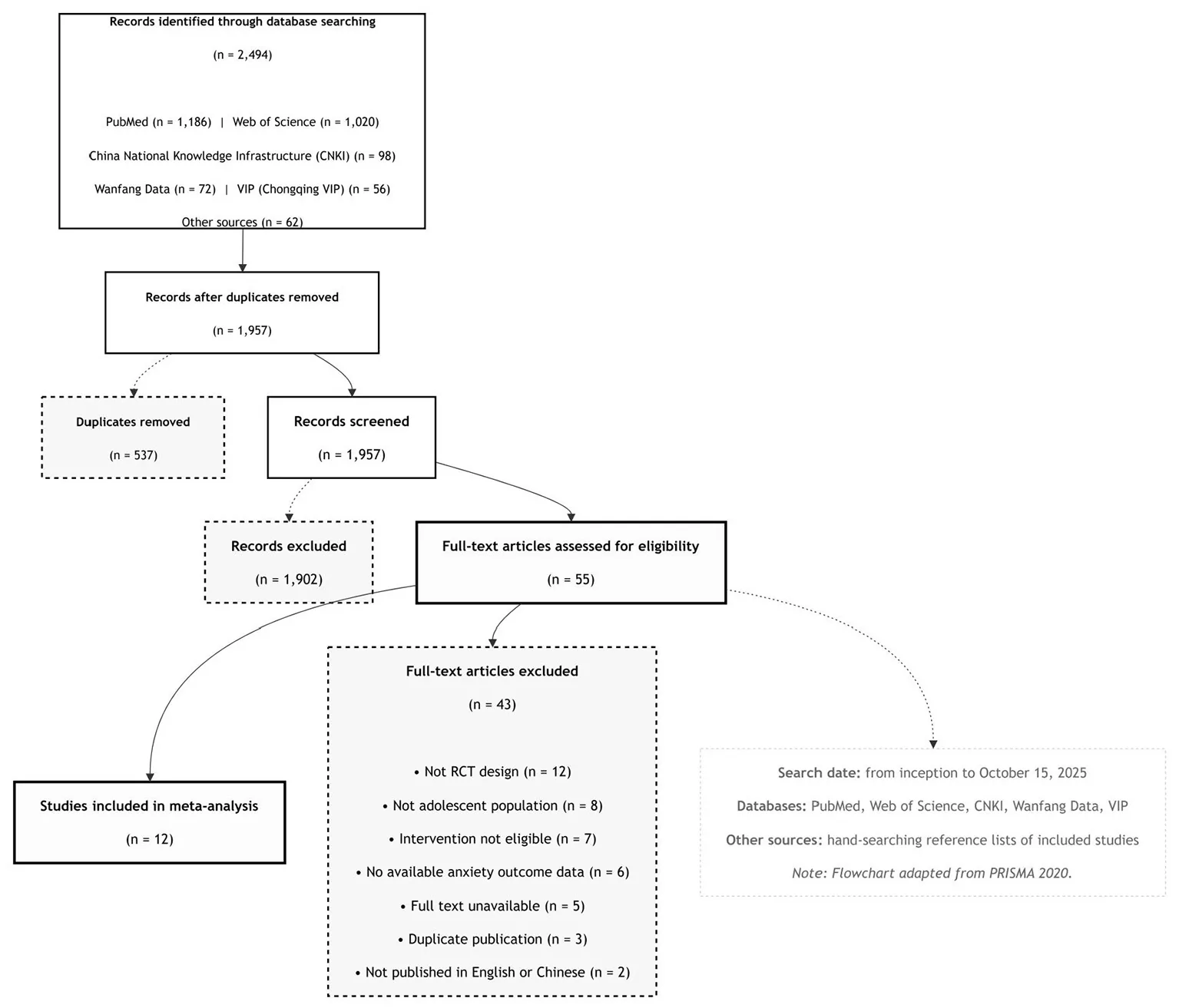
Flowchart of literature screening.

### 2.4 Data extraction and literature quality assessment

#### 2.4.1 Data extraction

To ensure the accuracy and reliability of the data extraction, three researchers were assigned to work together in this study. Two of them collaborated on the extraction of basic literature information and risk assessment, while the third researcher was responsible for the comprehensive verification of all the information extracted by the first two. If there are differences of opinion during the process, all members will reach a consensus through full consultation to ensure the accuracy of the data to the greatest extent.

#### 2.4.2 Evaluation of literature quality

This study strictly screened the included literature based on methodological quality. Referring to the Cochrane systematic review system, two researchers independently conducted the risk of bias assessment of the literature to jointly determine whether the literature ultimately qualified for inclusion in the meta-analysis.

### 2.5 Statistical processing

Data analysis was performed using Review Manager 5.4 software (Cochrane Collaboration, London, UK). Heterogeneity tests were first conducted on the included studies: if *I*^2^ ≥ 50% and *P* ≤ 0.1 suggested high heterogeneity among the studies, combined analysis was performed using the random-effects model; For the rest of the cases, fixed-effects models were used for analysis (Wei et al., 2022). After completing the model analysis, visually present the effect sizes of each study and the combined analysis results by drawing forest plots. Then, to identify the source of heterogeneity, further subgroup analyses and sensitivity analyses were conducted. Finally, a funnel plot was drawn to assess whether there was publication bias in this study. Registration Number of this study: CRD420251265936.

## 3 Results and analysis

### 3.1 Documentation characteristics

A total of 12 studies were included in this meta-analysis, with a total sample size of 1,874,951 in the experimental group, and 923 in the control group, respectively. The subjects were adolescents aged 10 to 19 years, and the intervention was mainly aerobic exercise. The basic characteristics of the included studies are detailed in Table 2.

**Table 2:** Basic characteristics of the included studies.

Studies	Countries	Age (years)	Sample size		Interventions		A single intervention time/min	Frequency/period	Outcome metrics
			T	C	T	C			
Bazzano et al. (2022)	USA	11–14	23	21	Yoga classes	Attention control	45 min	Once a week for 8 weeks	PHQA
Khalsa Sat Bir et al. (2012)	USA	15–19	74	47	Yoga classes	Physical education classes continue as usual.	30 min	Twice a week for 11 weeks	SRD
Noggle Jessica et al. (2012)	USA	17	36	15	Yoga therapy	Routine therapy	30 min	3 times a week for 10 weeks	POMS-SF
Bao and Kaimin (2015)	China	14.70 ± 0.7	80	80	Tai Chi Class	The 8th edition of Radio Calisthenics by the General Administration of Sport of China	60 min	5 times a week for 1 year (52 weeks)	PHCSCS
Bao (2013)	China	14.82–15.1	103	57	Tai Chi Practice	Do not practice Tai Chi	30 min	5 times a week for 1 year (52 weeks)	SCL-90
Philippot et al. (2022)	Australia	17.7	20	20	Aerobic training	Relaxation exercises	60 min	4 times a week for 6 weeks	STAI
Melnyk et al. (2013)	USA	14.70 ± 0.7	358	421	Aerobic training	Attention control	20 min	Once a week for 6 months (26 weeks)	HLBS
Jelalian et al. (2011)	USA	12.00 ± 0.9	44	45	Aerobic exercise	Companion activities	–	–, for 16 weeks	STAI
Melnyk Bernadette et al. (2009)	USA	15.50 ± 0.5	11	6	Aerobic exercise	Attention control	20 min	Once a week for 9 weeks	HLBS
Wang and Liu (2018)	China	14.00 ± 0.5	90	98	Aerobic exercise	Traditional physical education classroom teaching methods	45 min	4 times a week for 3 months (13 weeks)	GAD-7
Bonhauser et al. (2005)	USA	15.50 ± 0.9	98	100	Aerobic training	Basic training	30 min	5 times a week for 40 weeks	HADS
Robert-McComb et al. (2015)	The United States	19	14	13	Tai Chi	Breathing techniques	60 min	3 times a week for 8 weeks	STAI

–, Not reported.

### 3.2 Literature quality risk assessment

Methodological quality of included randomized controlled trials (RCTS) was evaluated using the Cochrane Risk of Bias tool. According to the criteria in the Cochrane manual, the risk of bias for each study was classified into three levels: “low risk,” “high risk,” or “questionable.” The assessment was conducted independently by two researchers. In case of disagreement, it was first resolved through mutual consultation and, if necessary, a third researcher was consulted to reach a consensus. Finally, based on the risk assessment results of all included studies, a bias risk map (Figure 2) and a literature quality assessment map (Figure 3) were drawn. This assessment not only systematically presents the methodological quality of the existing research evidence, but also provides an important basis for subsequent subgroup analysis and sensitivity analysis, helps to explore the potential impact of studies with different levels of risk of bias on the overall combined effect size, thereby ensuring the robustness and reliability of the conclusions of this study.

**Figure 2:**
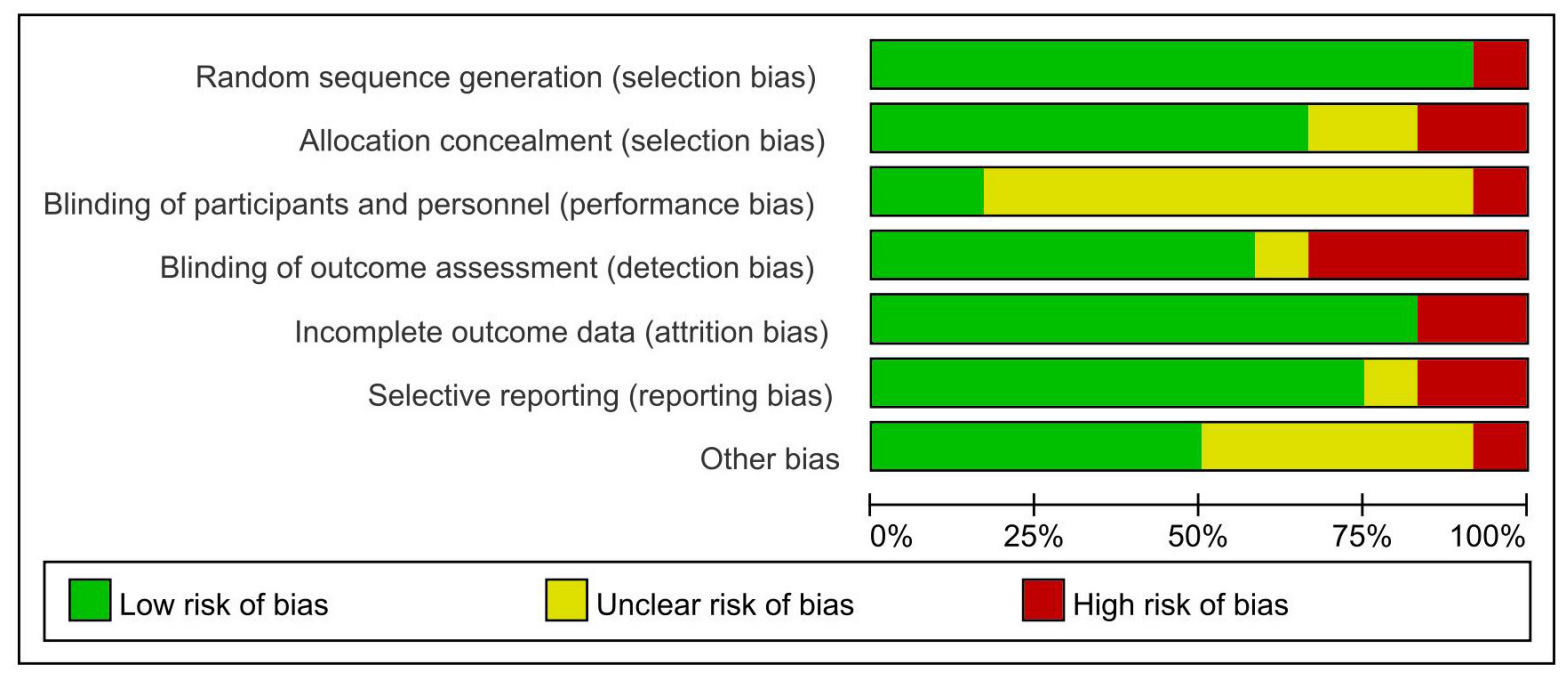
Summary of risk bias.

**Figure 3:**
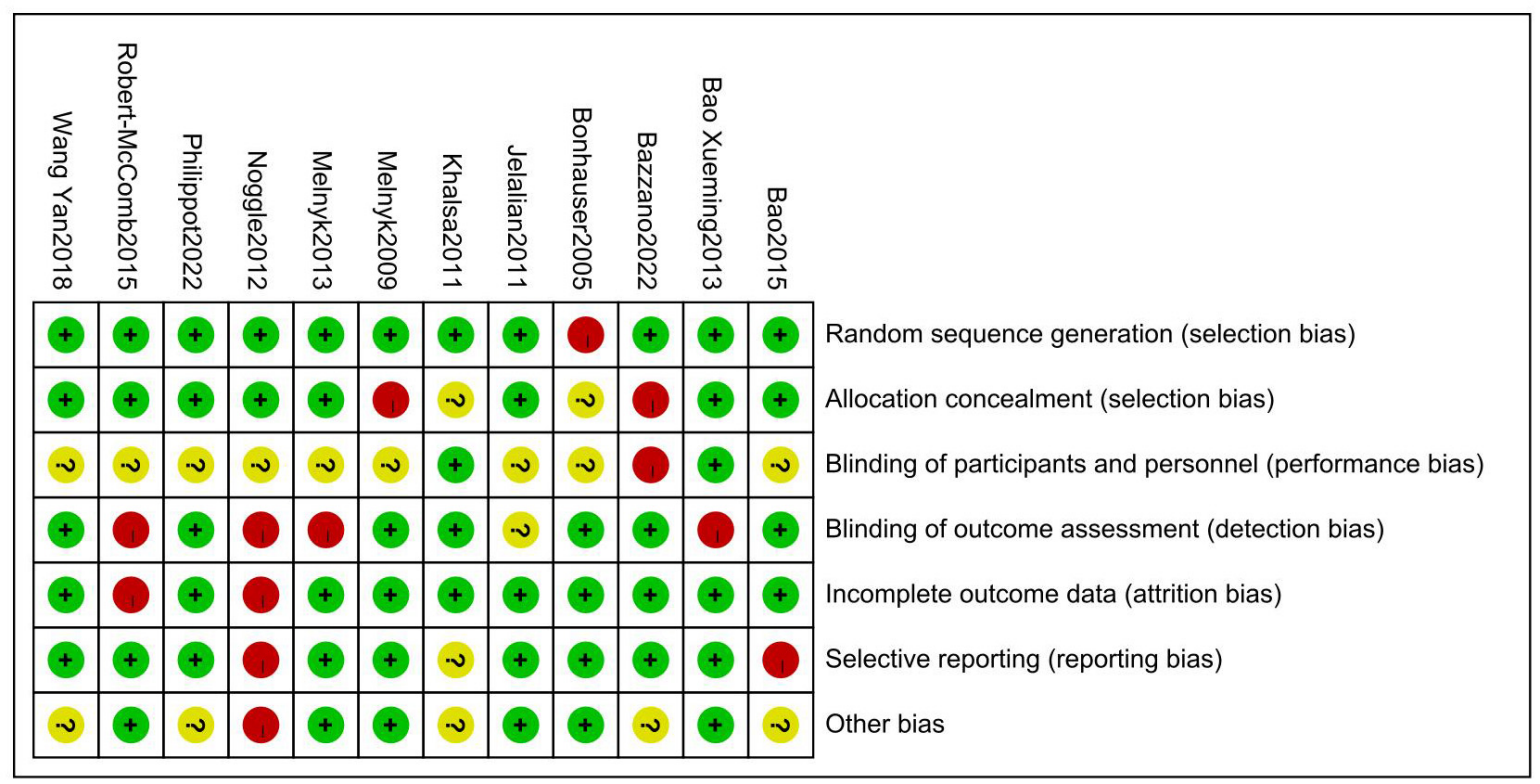
Evaluation of literature quality.

### 3.3 Overall effect of exercise interventions on anxiety symptoms in adolescents

This study selected status-Trait Anxiety Scale (STAI), Healthy Lifestyle Behavior Scale (HLBS), Self-Rating Personality Scale (SRP), Mood State Scale (POMS-SF), Adolescent Self-Concept Scale (PHCSCS), Symptom Self-Rating Scale (SCL-90), Generalized Anxiety Disorder Scale (GAD-7), and Hospital Anxiety The Depression Scale (HADS) was used as an outcome measure. The intervention effect size of exercise interventions (including aerobic exercise, Yoga, and Tai Chi) on anxiety symptoms in adolescents was analyzed using randomized controlled trials that met the inclusion criteria. Extract the relevant data from the literature and import it into Review Manager 5.4 software to calculate the standardized mean difference (SMD) and 95% confidence interval (95%CI) for the improvement of anxiety symptoms in adolescents by aerobic exercise.

The heterogeneity test for the included studies showed *I*^2^ = 87%, *P* < 0.00001, indicating substantial heterogeneity. A random-effects model was therefore used for the pooled analysis. The statistical results (Figure 4) showed that the overall effect size SMD of exercise interventions (pooling aerobic exercise, Yoga, and Tai Chi) on adolescent anxiety was −0.30, with a 95%CI of [−0.60, −0.01], *P* = 0.04. The high heterogeneity may be attributed to variations in exercise types, assessment instruments, intervention durations, and participant characteristics across studies (see Section 5.1 for detailed discussion). The statistical results (Figure 4) showed that the overall effect size SMD of exercise interventions (pooling aerobic exercise, Yoga, and Tai Chi) on adolescent anxiety was −0.30 (95% CI [−0.60, −0.01], *P* = 0.04). This result was significant, indicating that exercise-based interventions may reduce adolescent anxiety.

**Figure 4:**
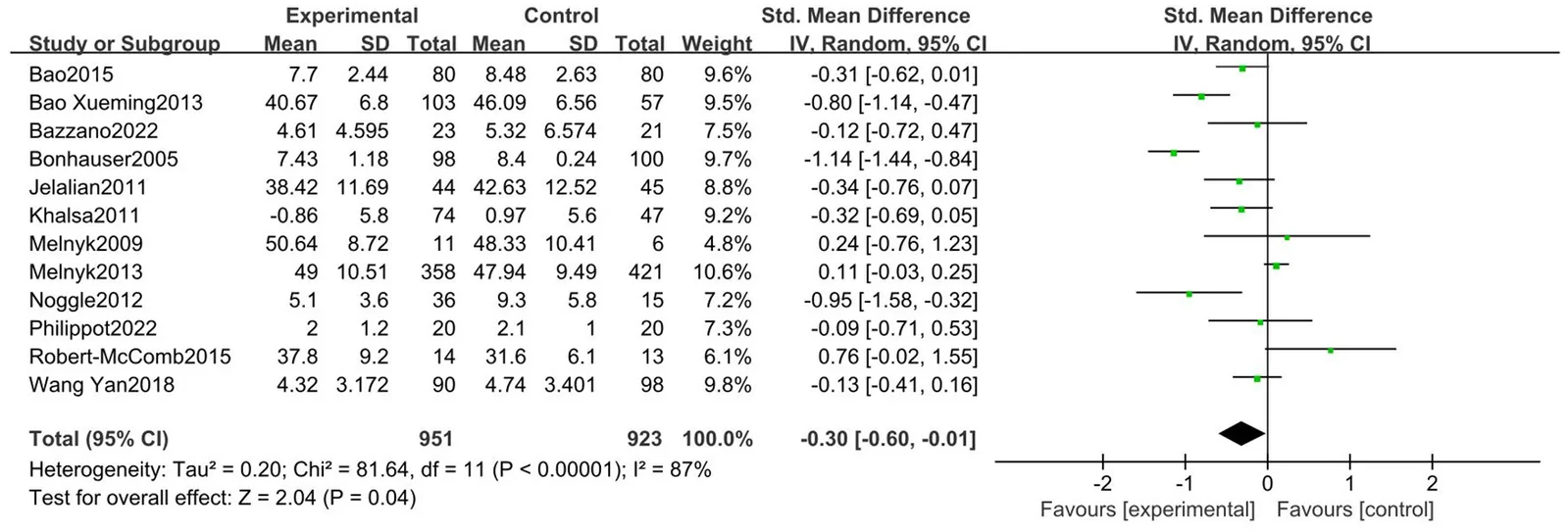
Forest plot of the overall effect of exercise interventions (including aerobic exercise, Yoga, and Tai Chi) on anxiety symptoms in adolescents.

### 3.4 Subgroup analysis

#### 3.4.1 Subgroup analysis of different sports interventions for adolescent anxiety symptoms

The intervention exercise items of the 12 included literatures were classified, and the intervention effects of yoga, Tai Chi and aerobic exercise on anxiety symptoms in adolescents were respectively, statistically analyzed (Figure 5). The intervention effects of the various exercise programs were highly heterogeneous (I^2^8 = 7%, *P* < 0.00001), indicating that aerobic exercise was an important influencing factor in the intervention of anxiety symptoms in adolescents. Subgroup analyses by exercise type showed that the Yoga intervention had a pooled effect size of SMD = −0.43 (95% CI [−0.85, −0.01], *P* = 0.14), which did not reach statistical significance. The Tai Chi intervention had a pooled effect size of SMD = −0.22 (95% CI [−0.87, 0.44], *P* = 0.52), which did not reach statistical significance. The aerobic exercise intervention had a pooled effect size of SMD = −0.26 (95% CI [−0.72, 0.20], *P* = 0.26), which did not reach statistical significance. Although none of the three individual subgroup effects reached statistical significance, it should be noted that the number of studies per subgroup was limited (Yoga: 3 studies; Tai Chi: 3 studies; aerobic exercise: 6 studies), and statistical power may have been insufficient. The overall pooled effect (see Section 4.3) reached statistical significance, which may reflect the cumulative effect of all individual study estimates.

**Figure 5:**
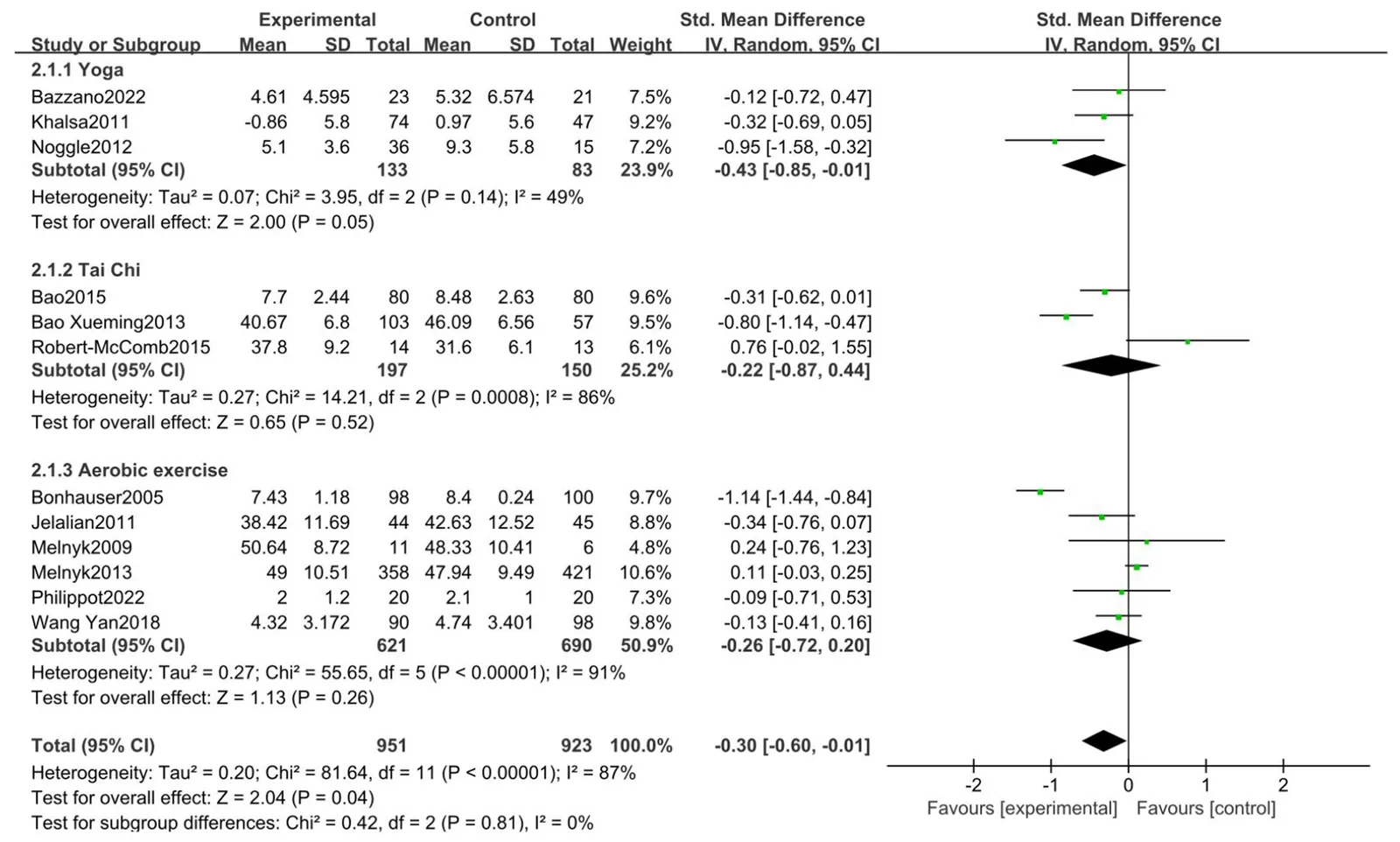
Meta-analysis forest plot of the intervention of anxiety symptoms in adolescents by different sports.

#### 3.4.2 Subgroup analysis of different single exercise durations on adolescent anxiety symptoms

The single exercise duration varies among the included studies, mainly divided into four groups: 20 min, 30 min, 45 min, and 60 min. Among the 12 studies, 2 studies had a single exercise duration of 20 min and 4 studies had a single exercise duration of 30 min. Two studies had a single session time of 45 min, three studies had a single session time of 60 min, and none of the studies had a single session time of any other group. Statistics on the intervention of anxiety symptoms in adolescents with different single exercise durations are shown in Figure 6. Among them, the effect SMD of 20-min exercise on anxiety symptoms in adolescents was 0.11, 95%CI [−0.03, 0.25], *P* = 0.13, indicating that 20-min exercise did not significantly intervene in anxiety symptoms in adolescents. The effect of 30 min of exercise on anxiety symptoms in adolescents was SMD=-0.80, 95%CI [−1.18, −0.42], *P* < 0.0001, indicating that 30 min of exercise could significantly intervene in anxiety symptoms in adolescents. The effect of 45 min exercise on anxiety symptoms in adolescents was SMD=-0.13, 95%CI [−0.38, 0.13], *P* = 0.34, indicating that 45 min exercise did not significantly intervene in anxiety symptoms in adolescents. The effect of 60 min of exercise on adolescent anxiety symptoms was SMD = 0.03, 95%CI [−0.53, 0.60], *P* = 0.91, indicating that 60 min of exercise did not significantly intervene in adolescent anxiety symptoms. Among the four session duration subgroups (20, 30, 45, and 60 min), only the 30-min session group showed a statistically significant effect (SMD = −0.80, 95% CI [−1.18, −0.42], *P* < 0.0001). However, this finding should be interpreted with caution, as only four studies were included in the 30-min subgroup analysis, and the number of studies in other duration subgroups was even smaller (ranging from 2 to 3 studies). These results are exploratory and do not establish 30 min as the optimal session duration.

**Figure 6:**
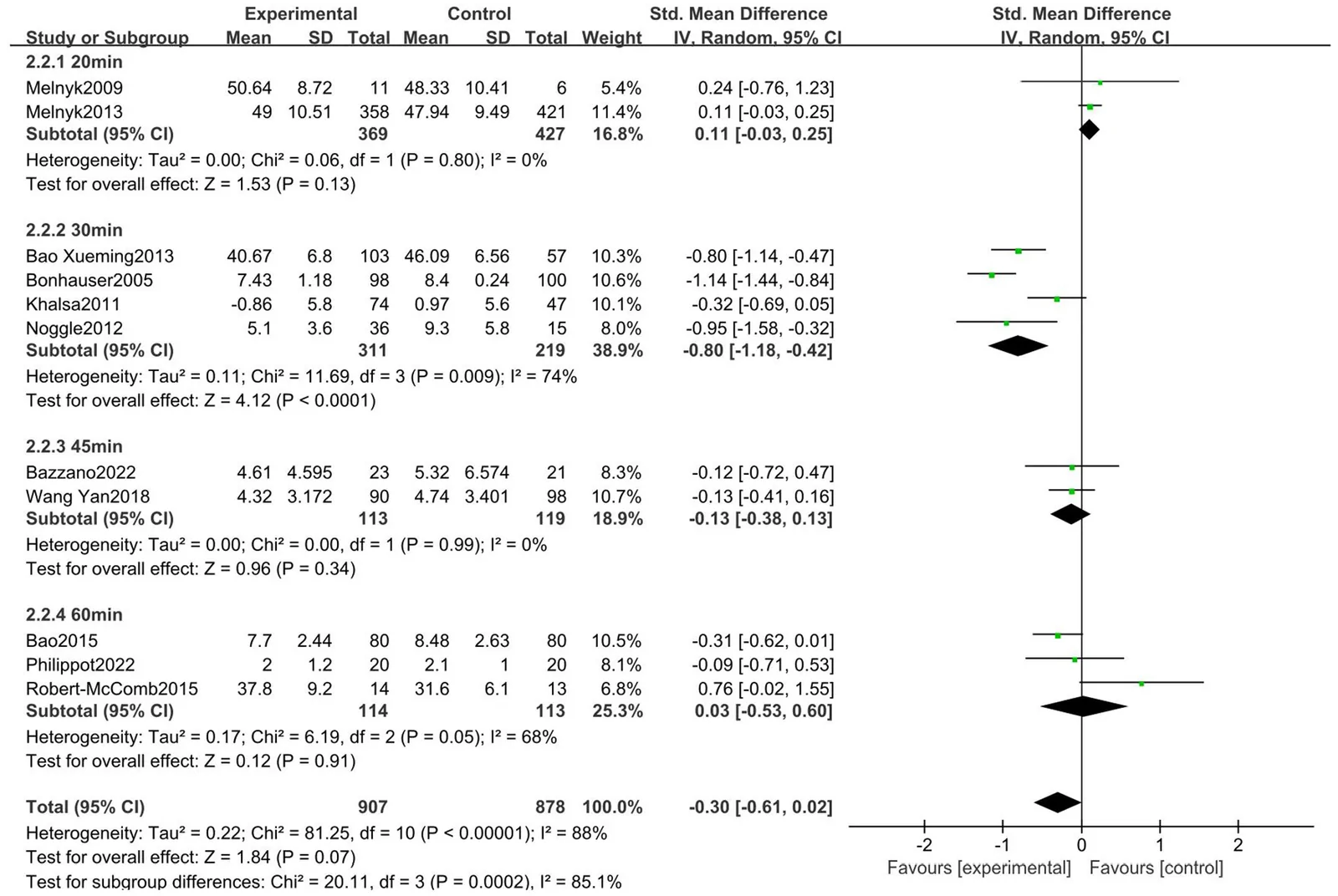
Meta-analysis forest plot of different single exercise durations on adolescent anxiety symptoms.

#### 3.4.3 Subgroup analysis of the intervention of anxiety symptoms in adolescents with different exercise frequencies

By counting the frequency of exercise interventions in each study, it was found that the frequency of exercise interventions ranged from once per week to five times per week. So the frequency of exercise intervention was set as 1 time/week, 2 times/week, 3 times/week, 4 times/week, 5 times/week, as shown in Figure 7. Among them, the effect SMD of once a week exercise intervention on adolescent anxiety symptoms was −0.03, 95%CI was [−0.54, 0.48], *P* = 0.91, indicating that once a week exercise frequency did not significantly intervene in adolescent anxiety symptoms. The intervention effect of twice a week of exercise was SMD = −0.32, 95%CI [−0.69, 0.05], *P* = 0.09, indicating that twice a week of exercise did not significantly intervene in adolescent anxiety symptoms. The intervention effect of 3 times a week of exercise was SMD = −0.11, 95%CI [−1.79, 1.57], *P* = 0.90, indicating that 3 times a week of exercise did not significantly intervene in adolescent anxiety symptoms. The intervention effect of 4 times a week for adolescent anxiety was SMD = −0.12, 95%CI [−0.38, 0.14], *P* = 0.36, indicating that 4 times a week of exercise did not significantly intervene in adolescent anxiety symptoms. The intervention effect of five times a week of exercise was SMD = −0.75, 95%CI [−1.24, −0.26], *P* = 0.003, indicating that 5 times a week of exercise could significantly intervene in adolescent anxiety symptoms. Among the five weekly frequency subgroups, the five-times-per-week group showed the largest effect size (SMD = −0.75, 95% CI [−1.24, −0.26], *P* = 0.003). However, this finding should be interpreted cautiously, as only two studies were included in this subgroup. The exploratory nature of this analysis precludes any definitive conclusion regarding optimal weekly frequency.

**Figure 7:**
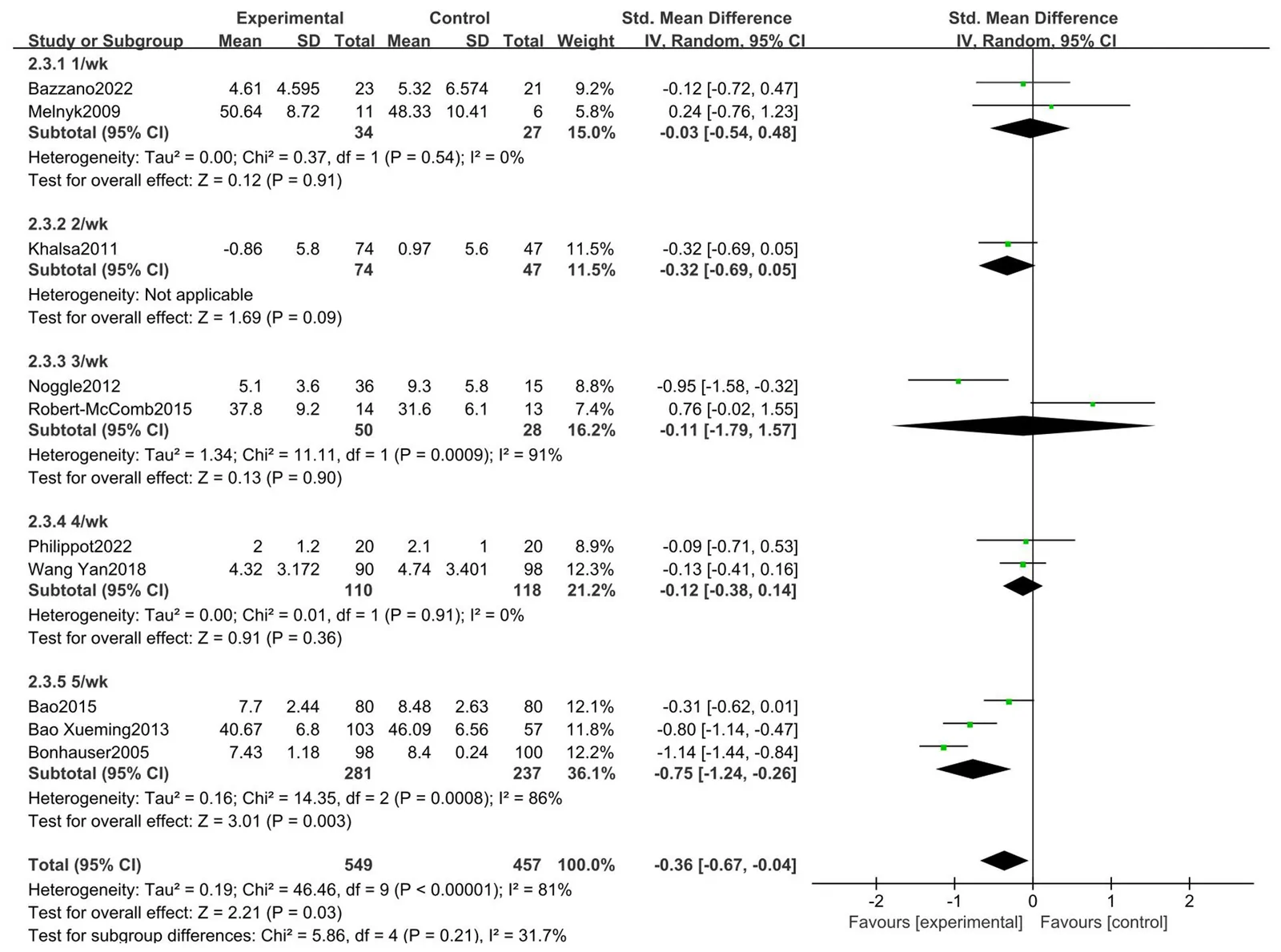
Meta-analysis forest plot of different exercise frequencies intervening in adolescent anxiety symptoms.

#### 3.4.4 Subgroup analysis of the intervention of anxiety symptoms in adolescents with different exercise cycles

Among the 12 included studies, those exploring the effects of short-term exercise intervention coexisted with those exploring the effects of long-term exercise intervention. There were significant differences in exercise intervention cycles among the studies. In this study, a 15-week cycle was used as a reference, and the effects of 15-week, 30-week, and 45-week exercise cycles on anxiety symptoms in adolescents were calculated respectively (Figure 8). Among them, the combined effect size SMD for a 15-week exercise cycle was −0.19, 95%CI was [−0.44, 0.06], *P* = 0.14, indicating that a 15-week exercise cycle did not significantly intervene in adolescent anxiety symptoms. The combined effect size SMD for a 30-week exercise cycle was 0.11, with a 95%CI of [−0.03, 0.25], *P* = 0.14, indicating that about 30 weeks of exercise did not significantly intervene in adolescent anxiety symptoms. The combined effect size SMD for a 45-week exercise cycle was −0.75, with a 95%CI of [−1.24, −0.26], *P* = 0.003, indicating that about 45 weeks of exercise could significantly intervene in adolescent anxiety symptoms. Among the three intervention duration subgroups, the 45-week cycle showed the largest effect size (SMD = −0.75, 95% CI [−1.24, −0.26], *P* = 0.003). However, this finding is based on only three studies (Bao and Kaimin, 2015; Bao, 2013; Bonhauser et al., 2005), all of which employed Tai Chi or aerobic exercise interventions with five-times-per-week frequency. Therefore, this result should be interpreted as exploratory and does not imply that 45 weeks is the optimal intervention duration.

**Figure 8:**
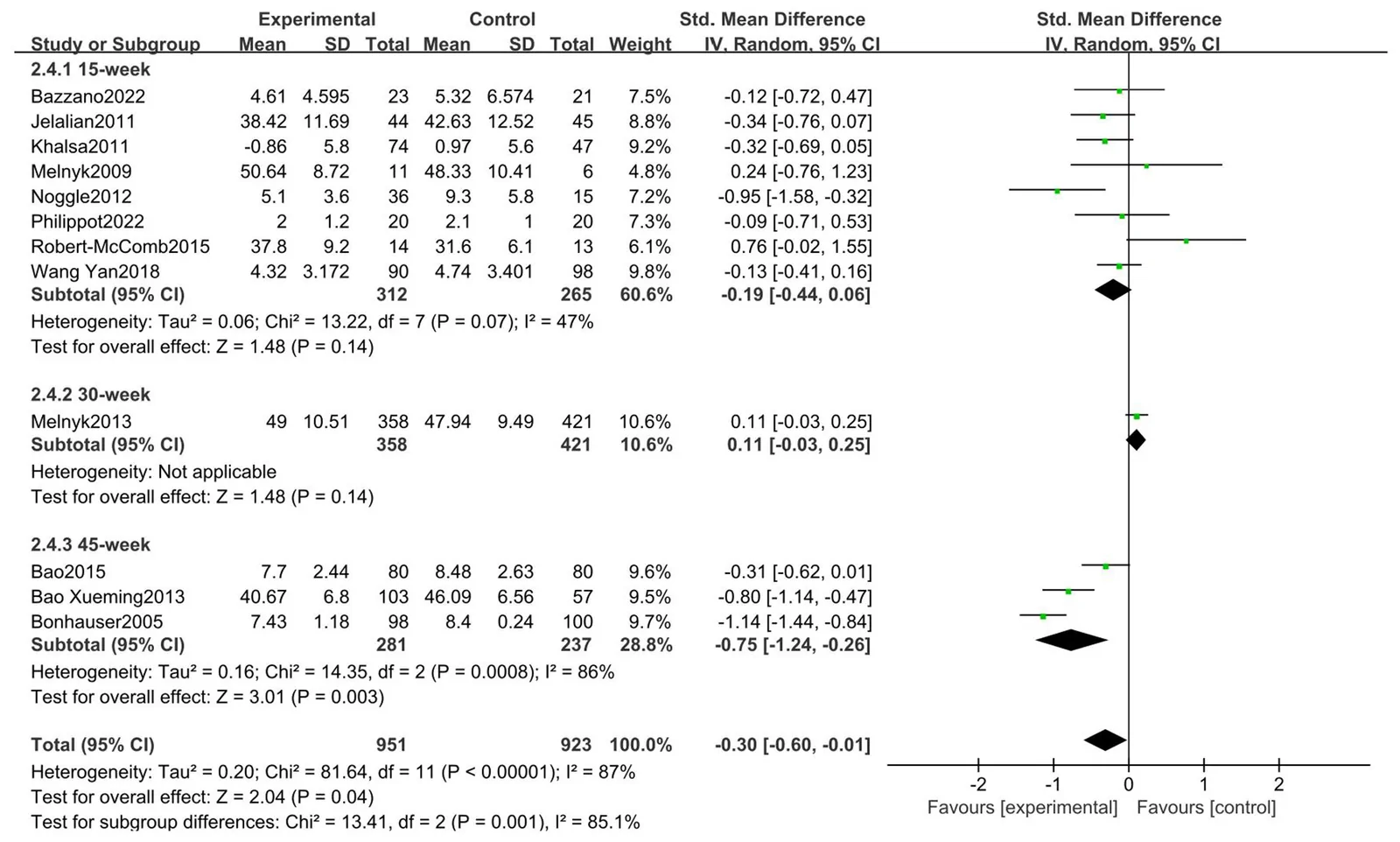
Meta-analysis forest plot of the intervention of anxiety symptoms in adolescents with different exercise cycles.

### 3.5 Sensitivity analysis

To test whether there is heterogeneity in the impact of individual studies on the overall study, a sensitivity analysis was conducted on the included literature, and the significance of the overall effect of aerobic exercise intervention on improving anxiety symptoms in adolescents was calculated by eliminating the literature one by one. Including all studies, the overall effect size SMD of exercise for improving anxiety symptoms in adolescents was −0.30, 95%CI was [−0.60, −0.01], *P* = 0.04, and the results were very significant. The results after excluding each article showed no significant changes in the meta-analysis results of the outcome measures after the included studies, indicating a high level of confidence in the meta-analysis.

### 3.6 Risk analysis of publication bias

Publication bias analysis was performed using Review Manager 5.4 software, and funnel plots were created for STAI, HLBS, SRP, PMS-SF, PHCSCS, SCL-90, GDA-7, and HADS outcome measures. The scatter was basically symmetrical, indicating that publication bias was not significant for STAI, HLBS, SRP, POMS-SF, PHCSCS, SCL-90, GAD-7, and HADS, as shown in Figure 9.

**Figure 9:**
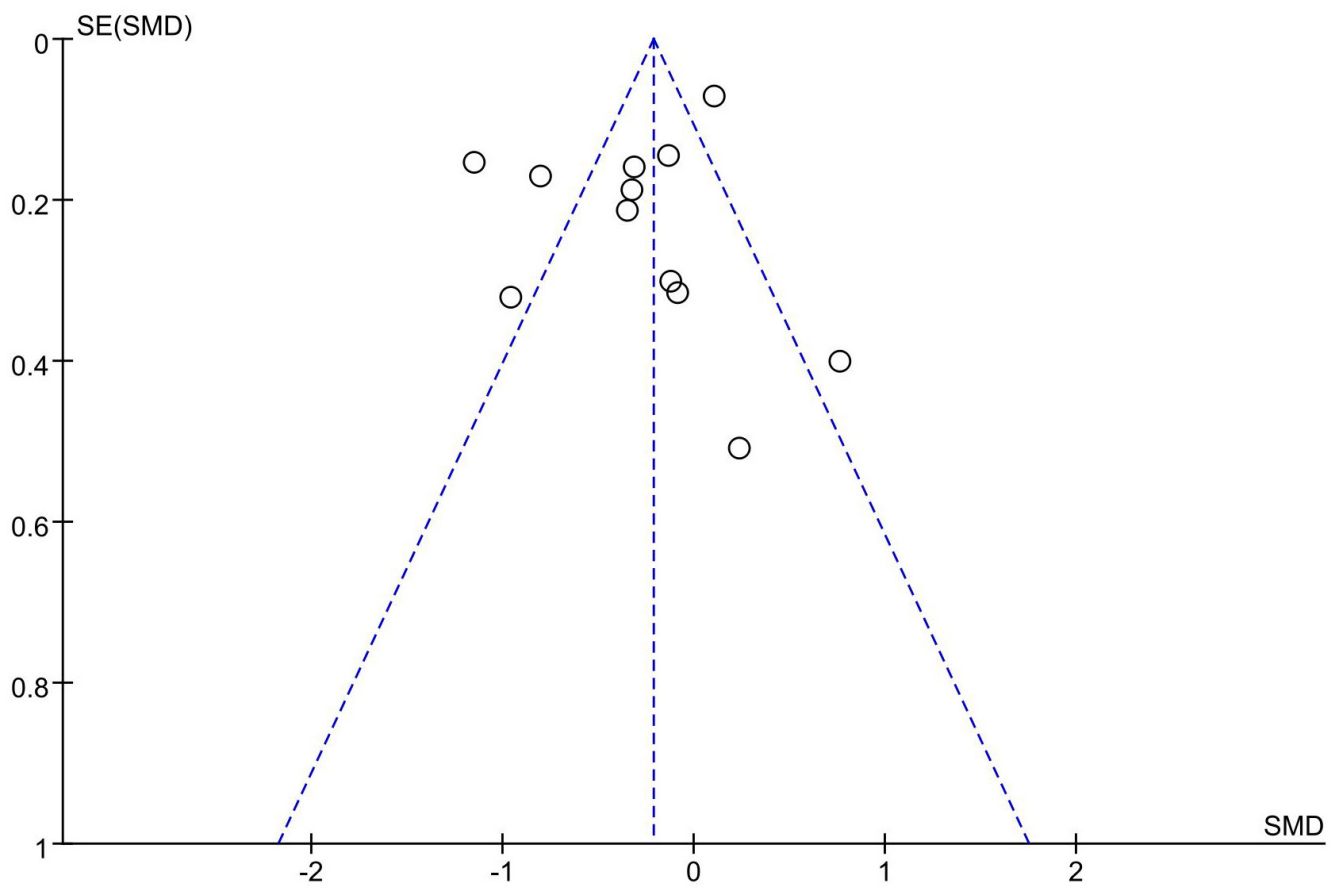
Funnel plot of publication bias in a meta-analysis of aerobic exercise intervention on anxiety symptoms in adolescents.

## 4 Discussion

### 4.1 Analysis of the overall intervention effect of exercise interventions on anxiety symptoms in adolescents

Adolescents are the core reserve force for the long-term development of the country, and their mental health status has always been one (Zhou et al., 2022) of the health issues that the World Health Organization focuses on. Previously, many studies have shown that aerobic exercise has the function of regulating mood and can effectively relieve anxiety symptoms. During exercise, the increase in serotonin levels in the body can balance the excitability of the nervous system and reduce mood swings; β -endorphins, as a natural analgesic and mood-regulating substance, can relieve physical fatigue and discomfort as well as help relieve mental tension in stressful situations; Dopamine, on the other hand, reinforces positive emotional experiences, providing psychological support for teenagers and helping them cope with stress challenges (Wang et al., 2024) more calmly. Regular aerobic exercise over a long period of time has a remarkable effect on enhancing neural plasticity in the brain. Studies show that regular exercise can significantly promote the synthesis of Brain-derived Neurotrophic Factor (BDNF). BDNF, a core protein that maintains the health and function of neurons, optimizes the efficiency of signal transmission between neurons. Elevated levels of BDNF not only enhance an individual's ability to regulate emotions but also have a positive effect (Azevedo et al., 2020) on social adaptation. Even short periods of moderate-to-high-intensity aerobic exercise can effectively enhance the psychological resilience of adolescents. Studies have found that just 30 min of moderate-intensity training can significantly reduce teenagers' overperception (LeBouthillier Daniel and Asmundson Gordon, 2015) of anxiety-related physiological responses such as accelerated heartbeat and shortness of breath.

Based on the findings, this study further focused on the adolescent group and systematically demonstrated the intervention effect of exercise (including aerobic exercise, Yoga, and Tai Chi) on their anxiety symptoms. Ultimately, 12 eligible experimental studies were included, with a total sample size of 1,874cases, including 951 in the experimental group and 923 in the control group, all of whom were adolescents. After the quality risk assessment of the literature, the overall quality of the included literature was high, indicating that the research results have reliable reference value. Based on the analysis of 12 studies, the anxiety symptoms of adolescents in the experimental group were relieved to varying degrees after different forms, frequencies and cycles of aerobic exercise intervention, confirming that exercise interventions can effectively improve the anxiety symptoms of adolescents. Due to the differences in the type of exercise, duration of intervention, frequency and cycle among different studies, subgroup analyses were further conducted in this study to identify the sources of heterogeneity.

### 4.2 Analysis of the effects of the exercise program intervention

The studies included in this review employed three types of exercise intervention programs: Yoga, Tai Chi, and aerobic exercise. Subgroup analyses revealed that the pooled effect sizes for all three types did not reach statistical significance (Yoga: *P* = 0.14; aerobic exercise: *P* = 0.26; Tai Chi: *P* = 0.52), which may be attributable to the limited number of studies in each subgroup (three, six, and three studies, respectively). Nevertheless, the direction of the effect sizes consistently favored exercise interventions, with Yoga demonstrating the largest effect (SMD = −0.43), followed by aerobic exercise (SMD = −0.26) and Tai Chi (SMD = −0.22).

Yoga, which combines slow voluntary body movements, full-body stretching and relaxation (Zou et al., 2018), abdominal breathing exercises, and concentrated meditation, is a typical mind-body exercise that may help reduce negative emotions while promoting physical and mental wellbeing (Liye et al., 2018). Previous studies have shown that Yoga can effectively regulate anxiety in older adults and play a positive role in their physical and mental health. In addition, a study of 210 college students demonstrated that practicing Yoga once a week for 12 weeks significantly alleviated anxiety among college students. Although the Yoga subgroup in our study showed the largest effect size among the three intervention types, it did not reach statistical significance (*P* = 0.14), likely due to the small number of included studies (*n* = 3). This finding should therefore be considered exploratory and requires confirmation through future high-quality research with larger sample sizes.

Aerobic exercise refers to rhythmic and sustainable physical activity that involves large muscle groups of the body with an adequate supply of oxygen (Zhengchun and Jian, 2021). In terms of alleviating anxiety, aerobic exercise has been ranked relatively high among all interventions. The ability of aerobic exercise to improve anxiety symptoms in adolescents can be explained by two hypotheses: the distraction hypothesis suggests that aerobic exercise can divert an individual's attention away from negative stimuli or physical discomfort (Jianping, 2013), thereby improving emotional state; the monoamine hypothesis suggests that aerobic exercise can enhance the transmission efficiency of aminergic synapses in the brain and increase the synthesis and release levels of monoamine neurotransmitters (Cui et al., 2023), thereby exerting a mood-regulating effect. Previous studies have confirmed that aerobic exercise, as an effective non-pharmacological intervention (Yu and Yunping, 2007), has a significant effect on improving negative emotions in adolescents. However, in our subgroup analysis, the pooled effect of aerobic exercise (SMD = −0.26) did not reach statistical significance (*P* = 0.26). This unexpected finding may be explained by the following factors: considerable methodological heterogeneity existed among the six aerobic exercise studies in terms of intervention intensity, frequency (Zhao, 2004), and duration; the sample sizes of the six studies varied widely (ranging from 19 to 779 participants), resulting in uneven statistical weighting; and different anxiety assessment tools were used across studies, which may have affected the standardization of effect sizes (Bao, 2008).

Regarding Tai Chi, although previous studies have shown its positive effects on both psychological and behavioral aspects (Song, 2014)—effectively alleviating negative emotions such as anxiety and depression—our subgroup analysis did not find a statistically significant effect (SMD = −0.22, *P* = 0.52). This may be due to the limited number of Tai Chi studies (*n* = 3) and the considerable variation in intervention protocols (e.g., session duration ranging from 30 to 60 min (Ke and Wang, 2022), frequency ranging from 3 to 5 times per week). Additionally, one of the included Tai Chi studies reported that the intervention focused more on movement and body form standardization rather than abdominal breathing training (Liu et al., 2024), which may have attenuated the mind-body benefits typically associated with Tai Chi practice (Ransford, 1982). These findings suggest that the effectiveness of Tai Chi for adolescent anxiety remains uncertain and requires further investigation with larger, well-designed trials (Wei, 2025). Importantly, none of the subgroup analyses by exercise type reached statistical significance (*P* > 0.05 for Yoga, Tai Chi, and aerobic exercise). This suggests that the significant overall effect (SMD = −0.30) is driven by the pooled estimate across all studies and does not provide evidence for the superiority of any single exercise type. The observed effect sizes (e.g., Yoga SMD = −0.43) are purely exploratory and require further investigation.

### 4.3 Analysis of the intervention effect of single exercise duration

Regarding the relationship between the duration of a single exercise session and anxiety symptoms in adolescents, relevant studies have shown that a daily exercise duration of no < 30 min is a key factor (Yong et al., 2007) in reducing the risk of anxiety among students. Moderate to high-intensity mixed exercise, more than three times a week for 20 to 60 min each time, for more than 12 weeks, is particularly effective in enhancing psychological stress resistance. Regular exercise interventions can not only effectively relieve tension in stressful situations such as exams, but also have positive effects that can last up to 40 weeks after the intervention ends (Zhang et al., 2023). At the same time, short periods of moderate-intensity aerobic exercise can also have a positive effect on the psychological resilience of teenagers. Studies have shown that just 30 min of moderate-intensity training can significantly reduce teenagers' hypersensitivity (LeBouthillier Daniel and Asmundson Gordon, 2015) to anxiety-related physiological signals such as accelerated heartbeat and shortness of breath, helping them to be more proactive in the face of anxiety. The study further found that among the literature included, only two studies set the duration of each session to 20 min, while the remaining 11 studies set the duration of each session to the range of 30 to 60 min. Among the four session duration subgroups analyzed (20, 30, 45, and 60 min), the 30-min session group showed the largest effect size (SMD = −0.80, 95% CI [−1.18, −0.42], *P* < 0.0001). This finding is consistent with previous research suggesting that daily exercise of no < 30 min may be a key factor in reducing anxiety risk among students. However, several important caveats should be noted. First, only four studies were included in the 30-min subgroup, and the number of studies in the other duration subgroups was even smaller (20-min: *n* = 2; 45-min: *n* = 2; 60-min: *n* = 3). Second, these four studies varied considerably in terms of intervention type (aerobic exercise, Yoga, and Tai Chi), frequency (ranging from one to five times per week), and total intervention duration (ranging from eight to 52 weeks). Third, we did not conduct a formal dose-response meta-regression to examine the continuous relationship between session duration and effect size. Therefore, the finding that 30-min sessions were associated with larger effect sizes should be considered exploratory rather than definitive. Future studies with standardized protocols and larger samples are needed to confirm the optimal session duration for reducing anxiety symptoms in adolescents.

### 4.4 Analysis of the effects of intervention on exercise frequency and cycle

Studies have shown that both the frequency of exercise and the intervention cycle have significant intervention effects on anxiety symptoms in adolescents. Teenagers showed a downward trend in anxiety levels after participating in aerobic exercise training at different frequencies. The experiment showed that the anxiety levels of the three groups decreased 2 weeks before the training, but there was no statistically significant difference. From the fourth week, as the frequency of exercise gradually increased, the decline in anxiety levels became apparent, with the high-frequency training group showing particularly significant intervention effects in the sixth and eighth weeks. The study also noted that the higher the frequency of exercise, the better the anxiety relief effect, suggesting that regular aerobic exercise over (Zhang and Li, 2024) a long period not only improves physical fitness but also helps adolescents better cope with anxiety. For teenagers with more obvious anxiety symptoms, consistent exercise intervention can gradually improve their emotional management ability and psychological resilience (Tao et al., 2023; Pang et al., 2023). The study set five exercise frequencies of once a week, twice a week, three times a week, four times a week, and five times a week. The results showed that five exercise frequencies per week had the best intervention effect (SMD = −0.75, 95%CI [−1.24, −0.26], *P* = 0.003); In terms of the intervention period, the study set up three groups at 15 weeks, 30 weeks and 45 weeks, with the 45-week intervention having a significant effect (SMD = −0.75, 95%CI [−1.24, −0.26], *P* = 0.003). A 5-week exercise frequency and a 45-week intervention cycle were both effective in improving anxiety symptoms in adolescents, which is consistent with the conclusions of the aforementioned study.

### 4.5 Risk of bias and certainty of evidence

Although the Cochrane Risk of Bias tool indicated that most included studies had low risk in domains such as random sequence generation and allocation concealment, several studies had high or unclear risk of bias in blinding of participants and personnel (performance bias) and blinding of outcome assessment (detection bias). Given the nature of exercise interventions, blinding of participants and instructors is inherently difficult, which may have introduced performance bias and potentially inflated the observed effect sizes. However, our sensitivity analyses (see Section 4.5) showed that excluding any single study did not significantly alter the overall pooled effect, suggesting that the main findings are not overly dependent on a single high-risk study. Nevertheless, the possibility that methodological weaknesses may have influenced the results cannot be ruled out, and readers should interpret the findings with this in mind.

Regarding the certainty of evidence, we did not perform a formal GRADE (Grading of Recommendations, Assessment, Development and Evaluations) assessment because the exploratory nature of our subgroup analyses and the small number of studies per subgroup precluded a reliable rating for most comparisons. However, based on the existing criteria—risk of bias, inconsistency (high heterogeneity, *I*^2^ = 87%), indirectness (use of diverse anxiety measures and inclusion of non-clinical samples), imprecision (wide confidence intervals and small sample sizes in subgroups), and potential publication bias (limited by the small number of studies)—the overall certainty of evidence for the primary outcome can be considered moderate to low. This suggests that while the intervention may be associated with a small reduction in anxiety, the evidence is not strong enough to support definitive clinical recommendations. Future large-scale, well-designed RCTs with standardized protocols are needed to improve the certainty of the evidence.

## 5 Limitations of the study

(1) The types of effect sizes used in this study are diverse and not unified, which may to some extent affect the consistency and comparability of the results integration; (2) Among the included studies, aerobic exercise literature was predominant, while yoga, Tai Chi and other exercise types accounted for a relatively small proportion, and the distribution of literature types was unbalanced, which may cause potential bias in the analysis results; (3) The intervention periods of the included studies varied greatly. To unify the analysis criteria, this study divided them into three groups nearby based on the length of the periods. This grouping method may have an impact on the accuracy of the research results. (4) We did not conduct a formal GRADE assessment due to the exploratory nature of the analysis and the limited number of studies, which limits our ability to confidently rate the certainty of the evidence. (5) Although we performed a risk of bias assessment, the results were not fully integrated into the interpretation of the overall effect due to the small number of studies; future updates with more trials should consider sensitivity analyses stratified by risk of bias.

## 6 Conclusions and recommendations

### 6.1 Conclusions

(1) This systematic review and meta-analysis suggests that exercise-based interventions may be associated with reductions in anxiety symptoms among adolescents (SMD = −0.30, 95% CI [−0.60, −0.01]), although the effect size is small and the evidence is constrained by substantial heterogeneity. Therefore, these findings should be interpreted with caution.

(2) Subgroup analyses indicated that exercise type (aerobic exercise, Yoga, and Tai Chi), session duration, weekly frequency, and total intervention duration may be moderating factors influencing intervention outcomes.

(3) Exploratory subgroup analyses showed that interventions with 30-min sessions, five times per week, and a total duration of approximately 45 weeks were associated with larger reductions in anxiety symptoms. However, due to the limited number of studies per subgroup and the absence of formal dose-response analyses, no definitive optimal exercise prescription can be established based on the current evidence. Future research with standardized protocols and larger sample sizes is warranted to validate these exploratory findings.

### 6.2 Recommendations

(1) It is recommended that educators and sports coaches develop individualized exercise prescriptions based on the gender, anxiety level, physical foundation and personal interests of adolescents, while taking into account the fun and sustainability of training, and avoiding resistance from adolescents due to excessive intensity or monotonous form of exercise. (2) It is recommended to use smart bracelets, sports watches and other devices to continuously monitor physiological indicators such as heart rate, resting heart rate and sleep quality of adolescents during the intervention process, using these objective and continuous physiological data as biomarkers for assessing anxiety levels to compensate for the recall bias that may exist in subjective scales. (3) Optimize the experimental design of the exercise intervention. Given the multiplicity of factors influencing adolescent anxiety symptoms, strict regulation and control of variables such as the life status, environment, personal experience and presence of bad habits of the research subjects should be carried out to reduce the interference of confounding factors on the results.
